# The state of mental health in Pakistan: analyzing system gaps and social determinants using data from the Global Mental Health Countdown 2030

**DOI:** 10.3389/fpubh.2026.1734392

**Published:** 2026-05-19

**Authors:** Shan Lateef, Alaa Sharaf, Tasnuva Shafin

**Affiliations:** 1 Department of Medicine, University of Oxford, Oxford, United Kingdom; 2 Department of Epidemiology and Biostatistics, University of Maryland, College Park, MD, United States; 3 Department of Medicine, SUNY Downstate Health Sciences University, Brooklyn, NY, United States

**Keywords:** Global Mental Health, health indicators, inequity, mental health systems, Pakistan, South Asia, low- and middle-income countries

## Abstract

**Objective:**

Mental health continues to be a neglected issue globally, and it is particularly disregarded in low- and middle-income countries (LMICs), where access to care is shaped by structural inequalities, pervasive stigma, and limited policy implementation. Here, we leverage data from the Global Mental Health Countdown 2030 to assess Pakistan’s mental health landscape with respect to 48 core indicators grouped into four domains: (A) social and environmental determinants, (B) demand and need for treatment, (C) quality of services provision, (D) wellbeing.

**Methods:**

Comparison groups were provided as medians for South Asia and the larger LMICs.

**Results:**

In relation to LMICs, Pakistan ranks in the bottom quartile for inclusiveness and displacement, and possesses increased concentrations of exposure to school bullying and pandemic-related anxiety. Psychological disorders are common and the treatment gap is over 90%, with much of care delivered by non-specialist or untrained providers. Moreover, decreased capacity levels in the system perpetuates mental health stigma. Although mental, neurological, and substance-use related disorders account for 6% of total DALYs, government spending on mental health is 0.4% of the budget in health. As a result, health outcomes are also reflected in low happiness scores and increasing rates of suicide. Psychosocial services are entirely absent at the primary care level, and systematic surveillance is nonexistent.

**Conclusion:**

This cross-sectional benchmark analysis underscores the urgent need for structural reforms, including primary care integration, investment in digital and community-delivered interventions, gender-responsive policies, and robust data systems to enable accountability and targeted policy action.

## Introduction

Mental health disorders are a significant and growing part of the burden of disease, and this impact is most severe in low- and middle-income countries (LMICs) where access to resources and treatments is limited and treatment gaps are common. In the paradigm of the global burden of disease, the mental and substance use disorders cause roughly 7.4% of all disability-adjusted life years (DALYs) globally, making them one of the leading causes of nonfatal burdens of illness worldwide (1). Importantly, even though this level of burden is so extensive, very little of the national health budget of LMICs tends to be spent on mental health care, and there are huge shortfalls of trained experts and access to treatment, creating huge treatment gaps (2, 3).

The challenges are compounded in countries like Pakistan by strong structural and sociocultural inhibitions. Stigmatization of mental illness is widespread, often founded on supernatural or moral explanations, and results in people avoiding seeking help (4). Mental health sector services are acutely inadequate, both qualitatively and by having one of the lowest proportions of mental health experts to the population within South Asia (5). A vast majority of psychiatric care occurs in urban tertiary hospitals, and there are significant shortages of trained staff and infrastructure in rural and remote communities (6, 7). For the national health budget of Pakistan, only fewer than 1% of financial allocations are assigned toward the field of mental health, and hence the growth of human resource capabilities and infrastructure gets limited (8).

Child and adolescent mental health services are also scarce, given the limited trained staff practicing beyond the provinces of Punjab and Sindh (9). Furthermore, school-based initiatives promoting mental health literacy are few; despite the emergence of new mobile initiatives, such as mPareshan, implementation remains limited in scope (10). Furthermore, dependencies on foreign aid, gender inequities, access to violence, and access inequities to healthcare and education all increase the proportion of mental illness among vulnerable populations (11).

To enhance global accountability and foster the comparison of data, the Global Mental Health 2020 (GMH2030) tracking system was designed, condensing measures relating to mental health across 193 states (12, 13). This program has the endorsement of various institutions, including United for Global Mental Health, Global Mental Health at Harvard, the Global Mental Health Peer Network, the World Health Organization, and the United Nations Children’s Fund. Through the United Nations, World Bank, and hundreds of academic partner databases, the dashboard assimilates national-level data relating to the infrastructure, workforce, financing, population well-being, and societal attitudes toward care. By aggregating this vast set of sources, the GMH2030 system provides an open database available to all, through which decision-makers, researchers, and advocates track and enhance mental health systems on the global stage (13).

The aim of this research is to consolidate Pakistan’s achievements on mental system strengthening and determine the key gaps in service coverage, policy, and social determinants according to the evidence of the Global Mental Health Countdown 2030 (GMH2030) dashboard. In addition, this paper contrasts the indicators of Pakistan with those of South Asian and LMIC comparators to reveal opportunities for improvement and enduring inequities. For this comparison, a cross-sectional benchmarking analysis was employed, based on GMH2030 data informed by peer-review literature. Sub-regional and global comparators, namely South Asia and LMIC medians, were utilized to put the performance of Pakistan into perspective. The intention is not to achieve the full or constituent-based coverage but rather an observational, evidence-based estimate of progress and gaps to inform policy and future research priorities.

## Materials and methods

The study replicates the approach used by colleagues in Greece to benchmark mental health indicators in Pakistan using the Global Mental Health (GMH) Countdown 2030 framework (14). The objective was to evaluate Pakistan’s performance across the GMH domains (A. Social and Environmental Determinants; B. Demand and Need for Care; C. Strength of the Mental Health System; D. Wellbeing), compare it to two comparator groups (South Asia and World Bank low-middle income countries), and identify policy-relevant gaps through quantitative ranking. The methods and variable definitions followed those previously outlined by Kotsis et al. (14).

### Data sources

Secondary data was drawn from the publicly available Global Mental Health Countdown 2030 dataset, which contains the GMH indicators, metadata, definitions, and sources (13). Supplementary data referenced within the GMH dataset (e.g., WHO Mental Health Atlas, World Bank, IHME) were used as documented (15, 16). At the time of extraction, the dataset contained 48 expected indicators.

### Comparator group rationale

Two comparable groups were identified to provide regional and economic context:

1. South Asia: Pakistan, India, Bangladesh, Nepal, Sri Lanka, Afghanistan, Bhutan, Maldives. Regional comparison highlights shared geographic, cultural, regional, and health system contexts.
2. Low-middle-income countries (LMICs): countries classified by the World Bank as lower-middle income. This economic benchmark offered perspective for comparable financial resources and health financing constraints.

The regional comparison contextualizes Pakistan’s results with neighboring countries, while the economic group comparison sets a benchmark for the country based on resources.

### Processing and indicator selection

All of Pakistan’s and the comparator group countries’ GMH indicators were extracted from the Countdown 2030 dataset, including the code, name, country, score, year, and data source (14). The indicator metadata was used to document high value indicators, flagging indicators where higher values indicated worse outcomes for inversion before the centile computation. Some indicators that were flagged for reversal included indicators for mental health disorder prevalence, substance use, and suicide mortality rates. The most recent and available metadata was pulled for all the indicators.

### Recoding indicators, score harmonization and reverse scoring

The GMH indicators included both categorical and ordinal measures. For categorical values a standardized 0–4 scale was applied with 0 being No/None/Absent, 1 being Limited/Minimal, 2 being Partial/Moderate, 3 being Good/Substantial and 4 being Excellent/Fully Implemented. These conversions were based on standardized groups within the GMH2030 dataset, reflecting the increasing levels of system strength and implementation. The scores were assigned to preserve the direction and order of the results. Numeric values were retained when available and used directly.

For indicators that indicated worse outcomes the scores were inverted so that all higher final scores indicated better performance. The reversed indicators were A.5.2, A.6.2, B.1.2, B.1.3, and B.1.4 as can be seen in Table 1. For the categorical scale, the inversion formula was: final score = 4 - raw score.

**Table 1:** Summary of Global Mental Health Countdown 2030 indicators for Pakistan across four domains.

Indicator	Indicator name	Pakistan	LMIC median	Centile	SA median	Centile
Domain A: Social and environmental determinants						
A.1.1	Inclusiveness Index	50.0	57.0	17.5	54.0	0.0
A.1.4	Women, Business and the Law Index	56.0	75.0	11.5	72.0	28.6
A.2.1	Unemployment, total (% of total labor force)	4.0	6.5	22.0	5.0	0.0
A.2.3	Poverty headcount ratio at $2.15 a day (2017 PPP) (% of population)	5.0	8.0	39.2	4.5	50.0
A.3.1	Prop. of students in school exposed to bullying	17.0	7.0	98.1	10.0	100.0
A.3.3	Mean years of schooling	5.0	7.0	5.8	7.0	14.3
A.4.1	# Internally Displaced People per 100,000 population	76.0	49.5	55.6	55.0	60.0
A.4.2	% Population that is made up of refugees in each country or territory of asylum	0.0	0.0	0.0	0.0	0.0
A.5.1	Average share of urban population within 400 m walking distance to an open public space (%)	38.0	47.5	27.3	38.0	33.3
A.5.2*	Mortality rate attributed to household and ambient air pollution, age-standardized (per 100,000 population)	115.0	90.0	78.8	101.0	85.7
A.6.1	Average COVID-19 Stringency Index	63.0	62.5	50.0	73.0	33.3
A.6.2*	COVID-19 pandemic-related excess anxiety and depression	2,029.0	1,296.0	76.5	1,950.0	57.1
Domain B: Demand and need for care						
B.1.1*	Age-standardized suicide rates (per 100,000 population)	10.0	8.0	53.8	6.0	57.1
B.1.2	Total alcohol consumption per capita (liters of pure alcohol, projected estimates, 15 + years of age)	0.0	3.0	0.0	1.0	0.0
B.1.3*	Age-standardized prevalence of mental disorders (% of population, 95% uncertainty interval)	13.0	12.0	71.2	12.0	57.1
B.1.4*	Age-standardized prevalence of substance use conditions (% of population, 95% uncertainty interval)	2.0	1.0	57.7	1.0	57.1
B.1.5	% total DALYs due to mental, neurological+substance-use	6.0	8.0	17.3	10.0	0.0
B.1.2	Total alcohol consumption per capita (liters of pure alcohol, projected estimates, 15 + years of age)	0.0	3.0	0.0	1.0	0.0
Domain C: Strength of mental health system						
C.1.1	Presence of a national stand-alone policy or plan for mental health	0.0	0.0	0.0	0.0	0.0
C.1.3	Extent to which the policy/plan complies with international human rights instruments	5.0	5.0	43.2	5.0	33.3
C.1.4	Extent to which the law complies with international human rights instruments	5.0	5.0	38.5	5.0	20.0
C.2.1	Total Mental Health Expenditure as a percentage of Total Health Expenditure	0.0	1.0	0.0	0.5	0.0
C.2.2	Development assistance for mental health	441.0	18.5	96.2	200.0	71.4
C.3.1	Total number of psychiatrists (per 100,000 population)	0.0	0.0	0.0	0.0	0.0
C.3.2	Total number of all other mental health professionals (per 100,000 population)	0.0	3.0	0.0	2.5	0.0
C.5.3	Psychosocial interventions for mental health conditions are available and provided at primary care level	0.0	0.0	0.0	0.0	0.0
C.6.1	Functional integration of mental health into primary care	3.0	3.0	23.7	3.0	33.3
C.7.1	Extent to which countries offer programs for promotion and prevention	2.0	2.0	45.7	7.0	20.0
Domain D: Wellbeing						
D.1.1	Happiness Ladder Score (0–10)	5.0	5.0	25.6	4.0	60.0

This table shows Pakistan’s performance across indicators of mental health determinants, system strength, service demand, and wellbeing, benchmarked against South Asian and LMIC medians. “Centile” values denote Pakistan’s relative rank within each comparator group—for instance, a centile of 39 means performing better than 39% and worse than 61% of countries. These reflect comparative, not population, measures. Indicators marked with an asterisk are reversed, where higher raw values indicated worse outcomes (e.g., prevalence or mortality) and were inverted so that higher centiles consistently represent better performance.

### Computation of ranks, centiles, and medians

Pakistan’s relative rank was computed using the percent_rank() function from the R dplyr package and was expressed as centiles (0–100). Comparator group medians were also computed, excluding Pakistan from each of the groups to provide a central tendency benchmark. Any missing data was flagged and excluded from the summary tables.

For visualization, the ggplot2 package was used to create a plot recording the centiles across the centile groups. Using the gt package a summary table was also created to indicate the code and name for each indicator, the values for Pakistan, the comparator medians, centile, year, and data source, grouped by the comparator group and GMH domain. Indicators where Pakistan fell to the bottom quartile (≤25th centile) were also compiled in order to prioritize policy recommendations.

### Missing data

The GMH2030 dataset originally contained 48 indicators across four domains. Of these, 29 indicators (60.4%) had complete, usable values for Pakistan after re-coding and converting the data. Nineteen indicators (39.6%) were excluded due to missing, non-numeric, and non-standard entries.

Missing data was addressed through a combination of recoding and indicator exclusion to ensure comparability across the countries. For categorical indicators, missing and non-standard textural responses were identified during recoding. Following this, only the values that matched the recoding criteria (e.g., None, Limited, Good, Fully Implemented) were converted into numeric scores. Any entries that did not match these categories, including blank and non-standard text, were coded as NA and retained as missing. For numerical indicators, only the values that were valid numbers were converted to numeric form. Values that were non-numeric or had empty entries were coded as NA.

During centile and ranking calculations, indicators with missing data were automatically excluded for rankings, medians, and summaries. Available-case analysis was utilized to compute medians using all non-missing comparator values. This method was used to align with standard practice for multi-country benchmarking analyses.

Indicators for Pakistan that had missing final scores were excluded from the centile visualization, domain level plots, and analysis. This ensures that the benchmarking and outputs reflect complete and interpretable data without artificially inflating or deflating Pakistan’s comparative position due to missingness.

To understand the impact of missing data on interpretation, we reviewed the distribution of missingness by domain. Consistent with global reporting patterns, the majority of the missing data was in Domain C, indicating gaps in data completeness for the strength of mental health systems. Indicators that had missing data for Pakistan included financing, service availability and workforce categories. Domain A, the Social and Environmental Determinants domain, also saw missing data, primarily for administrative reporting. Domain B, Demand and Need for Care, and Domain D, Wellbeing, had fewer data gaps.

### Software, packages, and reproducibility

All quantitative analyses were conducted in R version 4.5.1. Data management and wrangling were performed using tidyverse packages, including dplyr, tidyr, and stringr. Dplyr was used to manipulate data, tidyr was used to tidy the data, and stringr was used for string handling. Data was imported from Excel using readxl. Relative ranking and centile calculations were conducted using dplyr, which was again used for data manipulation. Tables were produced using gt, and visualizations were created using ggplot2.

## Results

This analysis assessed Pakistan’s standing across four domains of the Global Mental Health 2030 framework—(A) Social and Environmental Determinants, (B) Demand and Need for Care, (C) Strength of the Mental Health System, and (D) Wellbeing—using percentile-based comparisons against South Asian and low- and middle-income country (LMIC) benchmarks. Indicators were analyzed descriptively, with higher centiles reflecting comparatively poorer performance for adverse outcomes (e.g., poverty, bullying), whereas lower centiles reflect comparatively better performance. Findings are presented by domain, supported by corresponding tables and figures that illustrate Pakistan’s comparative position and key trends across metrics.

### Domain A: social and environmental determinants

**Figure 1:**
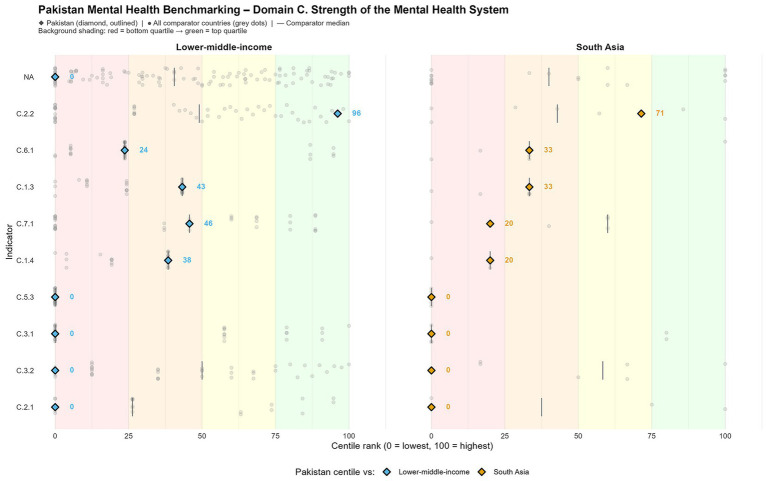
Social and environmental determinants of mental health: Pakistan vs. South Asia and LMIC medians.

Pakistan frequently stood toward the bottom 50% among both South Asian nations and LMICs, for Domain A (Figure 1). Specifically, the nation ranks on the 39th centile of LMICs by the poverty headcount ratio (A.2.3), indicating better performance than 39% of the nations and poorer than 61% (Table 1). Moreover, even by the ranking on the Inclusiveness Index, it falls on the 17th centile of LMICs.

Comparable vulnerabilities have been observed across various indicators. The prevalence of school bullying (A.3.1) is particularly alarming, positioning Pakistan in the 98th percentile among LMICs, indicating that student-reported instances of bullying exceed those in 98% of comparable nations (Table 1). Furthermore, displacement, quantified as the number of internally displaced persons per 100,000 individuals (A.4.1), was similarly elevated, ranking at the 56th percentile among LMICs (Table 1).

Unemployment rates (A.2.1) also signal economic vulnerability, and Pakistan falls at the 22nd centile of LMICs, indicating increased unemployment rates compared to most of these peers. Conversely, the country showed a relatively better performance on some reversed indicators: COVID-19 pandemic-related anxiety and depression incidence (A.6.2) at the 77th centile. This remark could be explained, in part, by poor surveillance and limited access to diagnostic services instead of genuinely lower prevalence of these conditions (14). These results may also suggest higher levels of communal and social support, specifically familial support which has been linked to psychological resilience during times of distress in Pakistan previously, and may contribute to improved relative outcomes as is seen with the A.6.2 indicator (17).

However, higher performance on pandemic-related anxiety and depression indicators may also demonstrate protective social factors, including family networks, community cohesion, and informal support systems that can compensate for crisis-driven psychological distress. Prior Global Mental Health work has found that collectivist social structures and reliance on extended support can attenuate mental health symptoms during population-level shocks, particularly in underserved settings (2, 12).

### Domain B: demand and need for care

Mental illness remains significantly prevalent in Pakistan. The age-standardized prevalence, which serves as an inverse indicator, positions the nation at the 71st centile among low- and middle-income countries (LMICs) regarding overall mental health conditions (B.1.3), suggesting a lower prevalence and, consequently, a comparatively superior performance. However, this observation could be due to an under-detection and under-reporting, rather than reflecting a genuinely reduced burden of disease, considering the limited availability of community-level screening and diagnostic capabilities (8, 18). This interpretation is supported by evidence demonstrating that Pakistan has among the lowest densities of trained mental health professionals in South Asia and lacks routine, standardized screening for common mental disorders at the primary care and community levels, resulting in substantial under-ascertainment of cases in population-level estimates (5, 19).

Moreover, differences in how psychological distress is managed may also shape observed patterns without demonstrating true differences in morbidity. In many settings, individuals may normalize distress, rely on informal or faith-based coping strategies, or delay formal help-seeking until symptoms become severe, which dampens measured prevalence despite ongoing impairment (4, 19). Thus, better-performing indicators may coexist with substantial unmet need, highlighting the importance of distinguishing observed demand from underlying burden when interpreting comparative rankings.

**Figure 2:**
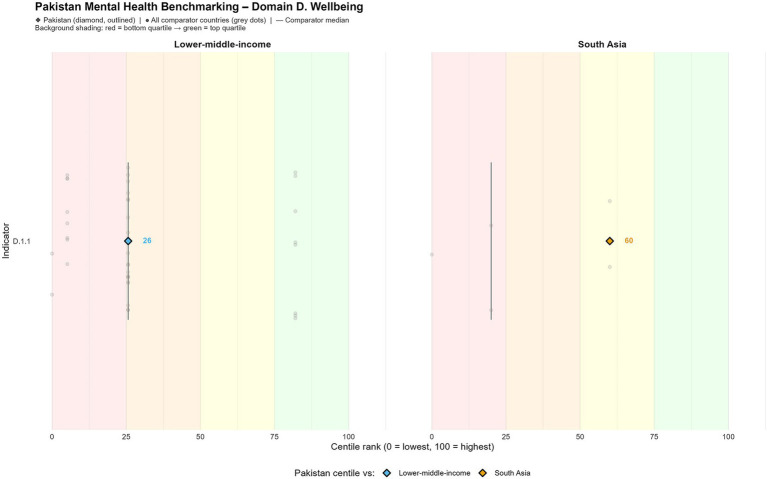
Treatment gap and burden of mental disorders in Pakistan compared to regional benchmarks.

Notably, suicide rates are situated at approximately the 50th centile when evaluated against both South Asian and LMIC standards (Table 1; Figure 2). Collectively, these patterns exemplify a contradiction of seemingly low prevalence alongside ongoing lethality, thus highlighting the discrepancy between reported mental health issues and the actual psychosocial distress experienced by the population (8).

### Domain C: strength of mental health system

**Figure 3:**
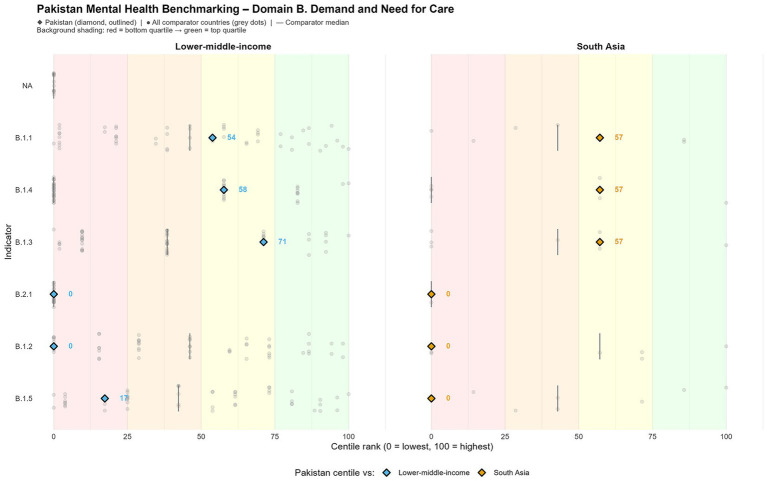
Mental health system capacity indicators in Pakistan.

Pakistan’s psychiatric system is still significantly underserved. Spending by the government on mental health constitutes only 0.4% of the national budget for health, and population coverage for mental health services is approximately 5% according to GMH2030 system coverage indicators derived from the WHO Mental Health Atlas (13). The proportion of psychiatrists to the population is extremely low, and just five psychiatric centers serve the colossal population of 180 million plus (Figure 3).

One of the particularly alarming disparities is the complete negligence of psychosocial services even in primary care, even though recommendations have continued throughout policy (5). This omission of provision points to challenges like shortage of workforce and political lethargy (20). However, recent digital interventions implemented by communities, like the mPareshan program, have shown acceptability and feasibility and have been successfully implemented by Lady Health Workers via home-based mobile counseling (10, 21). These rollouts are examples of the digital health solution holding much promise, even for underserved areas (8).

### Domain D: wellbeing

**Figure 4:**
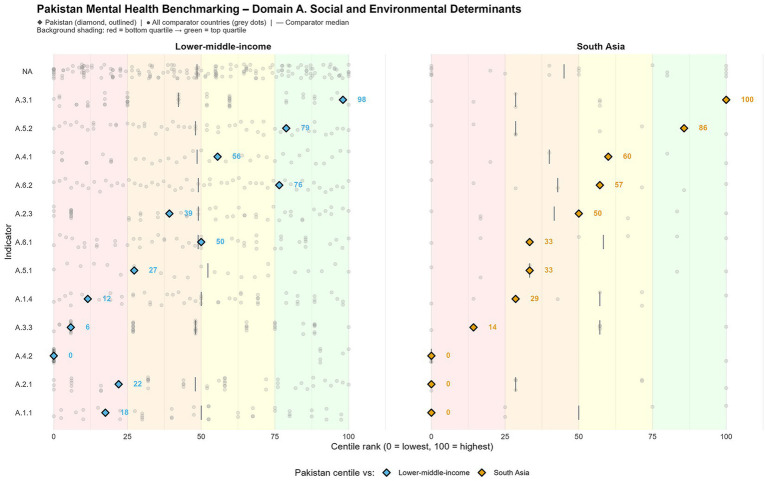
Wellbeing and population-level outcomes.

Health indicators are the resultant impact of insufficient investment and constrained accessibilities. Compared to the happiness score of 10, Pakistan lags behind both South Asia and nearby LMIC (Table 1; Figure 4). Suicide rates are most likely underreported due to societal stigma and reporting failure, but they are nonetheless on the rise, especially among females and youth (7). Persistent negative societal attitudes toward mental disorders are still hampering help-seeking behaviors and maintaining poor utilization of services rates (19).

## Discussion

The Global Mental Health Countdown 2030 indicators of the framework imply an under-performing, unequal and markedly under-funded mental health system. Across all the domains—social determinants, demand and need, system strength and wellbeing—Pakistan scores lower than the regional and LMIC median level (Table 1). These results echo years of evidence pointing to the mental health setting of Pakistan as one of centralized service delivery, financial under-investment and pandemic structural exclusion (8, 19). This comparative deficit elucidates how Pakistan’s impact has trailed behind regional peers such as India and Sri Lanka, both of whom have made substantial gains in integrating mental health into primary care and increasing workforce training (5, 6).

Factors associated with the social environment, such as poverty, gender inequality, and displacement, remain major drivers of mental illness. Winding these factors down would involve collective efforts beyond the healthcare sector, aligning policies in the spheres of education, employment, shelter, and social protections (12). Those jurisdictions which have successfully reduced the toll of mental illness have done so through aligning investments in healthcare alongside broader societal policies focused on the resolution of inequality—a strategic policy tack not yet uniformly adopted by Pakistan. Pakistan’s structural context is marked by urban–rural disparities, fragile governance, and recurrent humanitarian crises; mental health outcomes are thereby influenced by systemic exclusion from both economic and civic participation (7, 11).

The sizable treatment gap identifies a mismatch between population need and accessible services. Stigma (perceived and self stigma) toward mental illness, and cultural explanations for the supernatural cause of mental illness had caused late help-seeking and consequent low treatment uptake among these communities (19). Community-based public education programs, co-developed along with religious and cultural leaders, may help shift attitudes and make the delivery of mental health care more normative. This approach aligns closely with the GMH2030 priorities of parity between mental and physical health, community engagement, and development of culturally sensitive care methods (12).

Organizational-level availability of psychosocial services in primary care would likely be the most prominent barrier. Evidence elsewhere indicates the integration of psychiatric and psychosocial services within primary care delivery yields earlier detection, stigma minimization, and increased service utilization (22). The Lady Health Worker program outlines an existing structure delivering successfully to 85% of the rural Pakistani population, and this would be a viable means through which this integration would potentially be realized (10). Tele-psychiatry approaches and mobile intervention strategies have also offered scalable and financially sustainable avenues through which service delivery might be expanded (23). In all, such interventions illustrate how Pakistan could leverage GMH2030-aligned strategies to improve mental health systems despite fiscal limitations, operationalizing current infrastructures and digital tools (23, 24).

Ultimately, the lack of robust surveillance systems constrains the formulation of policies grounded in empirical evidence. Without regular national surveys, longitudinal data, or comprehensive metrics pertaining to mental health, policymakers will find it challenging to monitor the burden and assess interventions (25). Consequently, the establishment of a national mental health surveillance system is crucial for ensuring accountability, facilitating research, and informing program design. A key strength of this study is its systematic use of GMH2030 indicators to generate an internationally comparable baseline for Pakistan, filling a critical evidence gap in national mental health assessment and providing a platform for future evaluation.

### Limitations

Of the 48 indicators that were available in the GMH2030 dataset, only 29 (60%) had sufficient data for Pakistan to calculate a standardized score and centile. Of the 48 indicators, 19 (40%) were excluded due to missing or non-standardized values. Indicators with missing data could not be benchmarked against regional and income comparators, which reduced the total number of indicators available for comparison within each domain. Additionally, since the medians and centiles were calculated using available-case analysis, the comparator samples varied across the indicators. The indicators with higher missingness yielded less stable centiles and may have disproportionately reflected the results for countries with available data. Although this approach avoids assumptions for imputation, it does introduce variability in the precision of the centile estimates. To mitigate this, results were interpreted with attention to the data completeness, and the missingness was considered a measurement gap. Additionally, as a result of the missing data, domain-specific gaps may exist, and may bias the comparisons toward indicators with stronger global reporting infrastructure.

The GMH2030 framework also possesses structural limitations that constrain cross-national inference. The secondary dataset aggregates indicators drawn from heterogeneous sources with differing collection methods and reference years, which limits strict comparability across countries and over time (12, 13). Several indicators are based on sporadically updated surveys, conferring temporal misalignment that may influence regions with weaker reporting infrastructures. Moreover, the framework addresses quantifiable indicators and therefore does not encapsulate social drivers of health such as political instability, cultural norms, or informal care systems (14). As a result, observed cross-national differences should be interpreted as relative signals rather than precise estimates of underlying burden, and inferences regarding under-detection or apparent “better performance” must be made cautiously and in conjunction with contextual evidence.

It is also important to note that underreporting, especially as it pertains to mental health indicators such as suicide prevalence, is likely to be a constraint (26). Due to a combination of strong stigma, the criminalization of suicide, and religious beliefs regarding suicide, mental health issues data is likely to be underreported for mental health issues. As a result, some indicators may inaccurately represent the relative performance of the indicators.

Limitations also arise from the methods used when standardizing the indicators. While recoding the categorical indicators into the 0–4 ordinal scale ensured comparability across the countries, it reduces nuances and assumes that the categories were equidistant. For reversed adverse indicators, there was an assumption of linearity and that the higher values indicated worse performance, which may be an oversimplification. Additionally, since the percentile benchmarking reflects relative performance compared to peers it may mask reality. For instance, for indicators that present as stronger performers, underlying weaknesses may be hidden due to poor performance among comparator countries. Results cannot be interpreted at the subnational levels for that reason, and ecological fallacy may be of concern.

Additionally, GMH2030 itself has limitations. Although the dataset is comprehensive, the framework does not capture all relevant drivers of population mental health, including political instability, cultural factors, and health system quality. Some indicators also represented structural capacities, such as the availability of trained workforce, which may not directly translate to population level outcomes. For that reason, although the benchmarking represents relative strengths and weaknesses, it should not be taken as a definitive measure of the performance of Pakistan’s mental health system.

### Policy recommendations

Pakistan’s mental health crisis needs an evidence based and strategic response. One can identify several priority actions from the data:

- Inclusion of primary care of mental illness through the training of general practitioners and Lady Health Workers for the purpose of basic screening and counseling, aided by a system of referrals to higher specialized care.
- Utilize community-based and technological approaches, such as cell phone applications and telepsychiatry, to compensate for workforce shortages and engage populations that are hard to reach.
- Create a national system of surveillance on mental health using tailored indicators and frequent reporting to enable resource allocation and tracking of progress.
- Boost the investment in mental health to a minimum of 2% of the national health budget, and provide insurance cover for psychiatric disorders as well.
- Implement gender-sensitive, trauma-informed policies to avoid gender-based violence, adversity and displacement-related mental health risks.
- Initiate national anti-stigma and mental health literacy campaigns through engagement of religious and civil leaders to eliminate stigma related to seeking care and shift the views of people.

Overall, Pakistan’s mental health situation continues to be one of the most underdeveloped in the region. However, the alignment of data from GMH2030 and implementation research in recent years highlights some clear paths forward. By focusing on integrated primary care, community-level innovation, better data systems and more targeted policy reform, Pakistan can start to close the mental health treatment gap and set the foundations for a fairer and more resilient mental health system.

## Data Availability

Publicly available datasets were analyzed in this study. This data can be found at: Original source: https://data.unicef.org/resources/countdown-for-global-mental-health-2030-dashboard/; Direct data: https://zenodo.org/records/17465743.
